# Study of *micro-trichome* (*mict*) reveals novel connections between transcriptional regulation of multicellular trichome development and specific metabolism in cucumber

**DOI:** 10.1038/s41438-020-00456-0

**Published:** 2021-02-01

**Authors:** Jian Pan, Leyu Zhang, Guanqun Chen, Haifan Wen, Yue Chen, Hui Du, Junlong Zhao, Huanle He, Hongli Lian, Huiming Chen, Jianxin Shi, Run Cai, Gang Wang, Junsong Pan

**Affiliations:** 1 School of Agriculture and Biology, Shanghai Jiao Tong University, Shanghai, 200240 China; 2 School of Design, Shanghai Jiao Tong University, Shanghai, 200240 China; 3 Hunan Vegetable Research Institute, Hunan Academy of Agriculture Sciences, Changsha, 410125 China; 4 Joint International Research Laboratory of Metabolic & Developmental Sciences, School of Life Sciences and Biotechnology, Shanghai Jiao Tong University, Shanghai, 200240 China; 5 State Key Laboratory of Vegetable Germplasm Innovation, Tianjin, 300384 China

**Keywords:** Gene regulation, Secondary metabolism, Plant molecular biology

## Abstract

Trichomes that cover the epidermis of aerial plant organs play multiple roles in plant protection. Compared with a unicellular trichome in model plants, the development mechanism of the multicellular trichome is largely unclear. Notably, variations in trichome development are often accompanied by defects in the biosynthesis of cuticle and secondary metabolites; however, major questions about the interactions between developmental differences in trichomes and defects in metabolic pathways remain unanswered. Here, we characterized the glabrous mutant *mict*/*csgl1/cstbh* via combined metabolomic and transcriptomic analyses to extend our limited knowledge regarding multicellular trichome development and metabolism in cucumber. *Mict* was found to be explicitly expressed within trichome cells. Transcriptomic analysis indicated that genes involved in flavonoid and cuticle metabolism are significantly downregulated in *mict* mutants. Further metabolomic analysis confirmed that flavonoids, lipids, and cuticle compositions are dramatically altered in *mict* mutants. Additional studies revealed that *Mict* regulates flavonoid, lipid, and cuticle biosynthesis by likely directly binding to downstream functional genes, such as *CsTT4*, *CsFLS1*, *CsCER26*, and *CsMYB36*. These findings suggest that specific metabolic pathways (e.g., flavonoids and cuticle components) are co-regulated by *Mict* and provide insights into transcriptional regulation mechanisms of multicellular trichome development and its specific metabolism in cucumber.

## Introduction

Plant trichomes are highly specialized structures located on the surfaces of aerial organs. They play essential roles in diverse biotic and abiotic adaptation, for instance, deterring insects, herbivores, and microbes; secreting ions and contaminated metals^1^; reducing mechanical wear; regulating surface temperature; collecting and dispersing pollen; and absorbing water and nutrients^2^. Morphologically, trichomes can be glandular trichomes or non-glandular trichomes, unicellular or multicellular, and branched or unbranched. In addition, they have become an excellent model system for the analysis of cell differentiation as they are easily accessible in model plants such as *Arabidopsis thaliana*^3^. Trichome development was reported to have a close relationship with increased metabolite levels, such as flavonoids, anthocyanins, and phenylpropanoids^4^.

In the model plant *Arabidopsis* with non-glandular and unicellular trichomes, expression maps of leaf trichomes revealed high anthocyanin and flavonoid pathway activities, indicating the roles of trichomes in secondary metabolism and defense^4^. Similarly, in *Brassica rapa* with non-glandular trichomes, overexpression of *BraLTP2* leads to increased trichome density and affects the accumulation of secondary metabolites^5^. In *Solanum lycopersicum L*., *jasmonic acid–insensitive1* (*jai1*) is defective in JA signaling, and there was a significant reduction in the density and type of trichomes and monoterpene levels in the *jai-1* mutant^6^. In addition, Trichomes in the *hairless* (*hl*) mutant show abnormal morphology and density, reduced sesquiterpene and polyphenolic compounds, and increased susceptibility to insects and pathogens^7^. In addition, *SlCycB2*, which encodes a B-type cyclin gene, is involved in the initiation of multicellular trichomes and terpenoid biosynthesis. Nevertheless, little is known about the developmental process and underlying regulatory molecular mechanisms of multicellular non-glandular trichomes.

Cuticles are composed of polymer matrix cutin that is covered and embedded with waxes, and both are synthesized by underlying epidermal cells. Cutin is a biopolyester composed of C16 and C18 hydroxy and epoxy fatty acids, while wax is a complex mixture of very long chain fatty acids (VLCFAs), predominantly of chain lengths from 26 to 34 carbons. Nevertheless, wax also includes secondary metabolites and triterpenoids, such as flavonoids and sterols^8^. Wax aliphatic compounds are produced via the alcohol-forming and alkane-forming pathways^9^, leading to VLC alkanes and their derivatives^10^. *eceriferum* (*cer*) mutants present abnormal wax compositions or constituents in *Arabidopsis thaliana*^11^. According to mutant phenotype expression patterns and heterologous expression in yeast, *CER6* participates in producing fatty acids with 26 and 28 carbons^12^. Notably, it has been proven that *CER26* has a major role in C30 elongation, with high tissue and substrate specificity^13^.

Cuticles are also special structures on the epidermis of aerial plant organs. However, different from trichomes, which are generally considered “biofactories” for producing valuable natural products, the cuticle is the interphase between the plant and its surrounding environment^14^. In *Artemisia annua*, a novel HD-ZIP IV/MIXTA complex plays a significant role in regulating epidermal development, including cuticle formation and glandular trichome initiation^15^.

In cucumber, trichomes are multicellular, unbranched cells that are regularly distributed on most aerial surfaces. *tril/csgl3* and *csgl1/mict* are important cucumber mutants that enable the dissection of trichome development into distinct, genetically controlled steps, as follows: *Trichome-less* (*Tril*) regulates the initiation and density of the trichome and *Micro-trichome* (*Mict*) allows trichomes to differentiate correctly^16,17^. Both *Tril* and *Mict* are HD-ZIP transcription factors, different from the well-known MYB-WD40-bHLH complex in *Arabidopsis*. Unlike the unicellular trichomes in *Arabidopsis*, trichomes in cucumber allow cells to divide and to form into specialized trichome cells, such as fruit spines and fruit tumors. The regulatory network for the formation of trichomes and its relevance to cuticles in cucumber remains largely unknown.

In a previous study, we found that *Mict* regulates trichome initiation^17^. In this study, we investigated the underlying metabolomic and transcriptomic changes in *mict* mutants and uncovered *Mict* regulatory pathways in the cuticle and secondary metabolism. The results provide valuable molecular information for further studies on cucumber fruit external quality traits related to market value.

## Results

### *Mict* loss of function mutants exhibit profoundly altered leaf and fruit epidermis properties

**Fig. 1:**
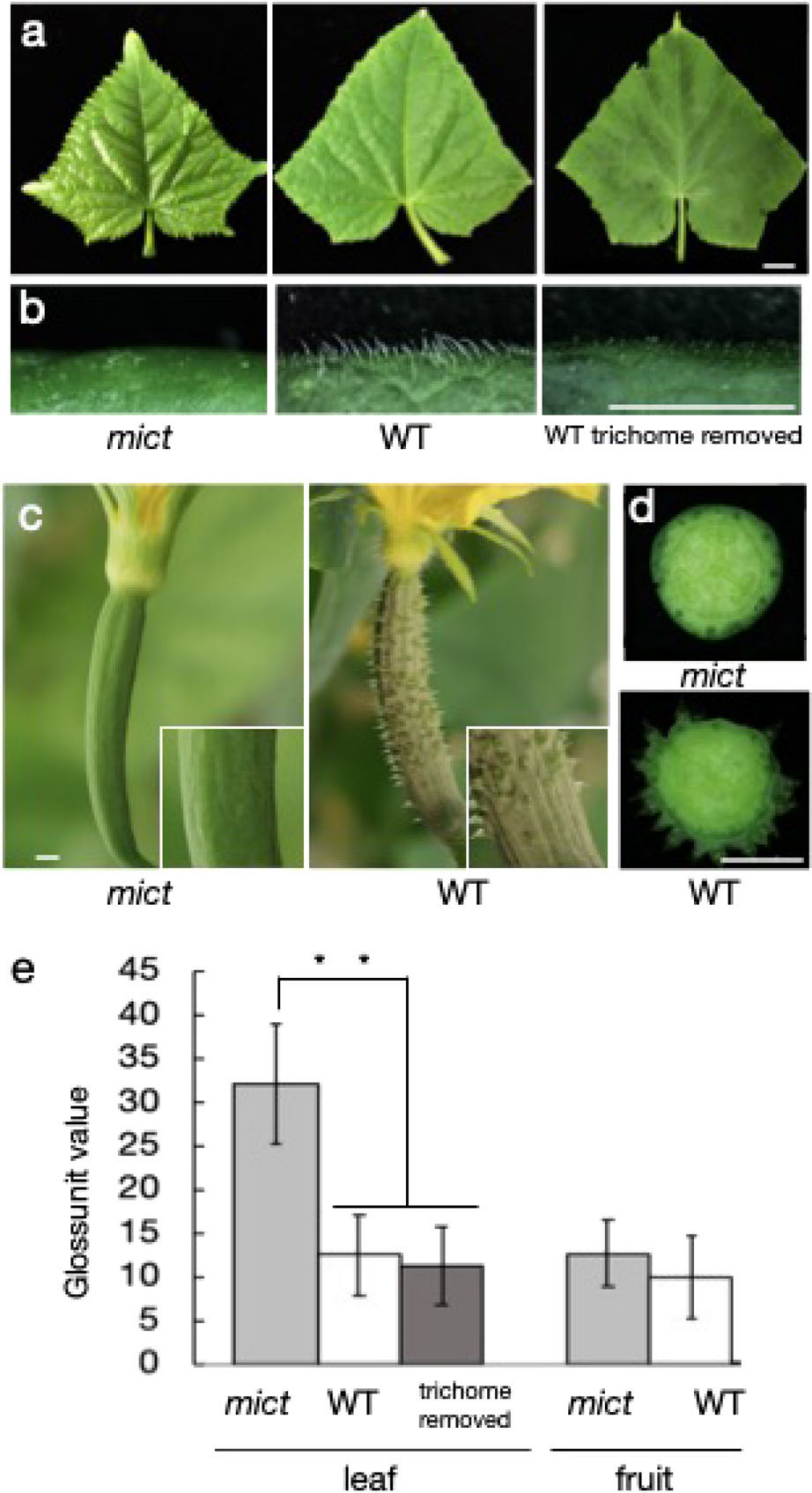
Phenotypic analysis of *mict* mutants and wild-type cucumber plants. **a** Phenotype of *mict* mutant and wild-type (WT) leaves as well as WT leaves with trichomes removed (from left to right). **b** The leaf surfaces corresponding to (**a**). **c** Phenotype of *mict* mutant and WT fruits. **d** Cross-section of *mict* mutant and WT fruits. **e** Gloss unit value of leaves and fruits from the *mict* mutant and WT. The asterisks represent significant differences between samples (Student’s *t*-test, ^**^P < 0.01, *n* = 10)

*Mict* has previously been reported to be involved in the development of trichomes in cucumber fruits and leaves^17^. However, many other profound phenotypic changes were found in *mict* mutants; for example, mutant leaves looked brighter and glossier (Fig. 1a, b) and mutant fruit surfaces were smooth (Fig. 1c). However, the *mict* mutant sarcocarp did not show visible phenotypic differences in color, gloss, or cell morphology compared with wild-type (Fig. 1d). Gloss levels (gloss unit value) of the leaves and fruits were examined by a gloss meter, which showed that *mict* leaf but not fruit surfaces were glossier than those of the WT (Fig. 1e). The gloss levels of the *mict* leaves were significantly higher than those of WT leaves even when trichomes were removed artificially, indicating that the gloss unit value is not affected by the presence or absence of trichomes. This result implied that mutation of *Mict* affects not only epidermal properties, but also epidermal cell metabolism in cucumber.

### *Mict* expression is highly associated with trichome development

**Fig. 2:**
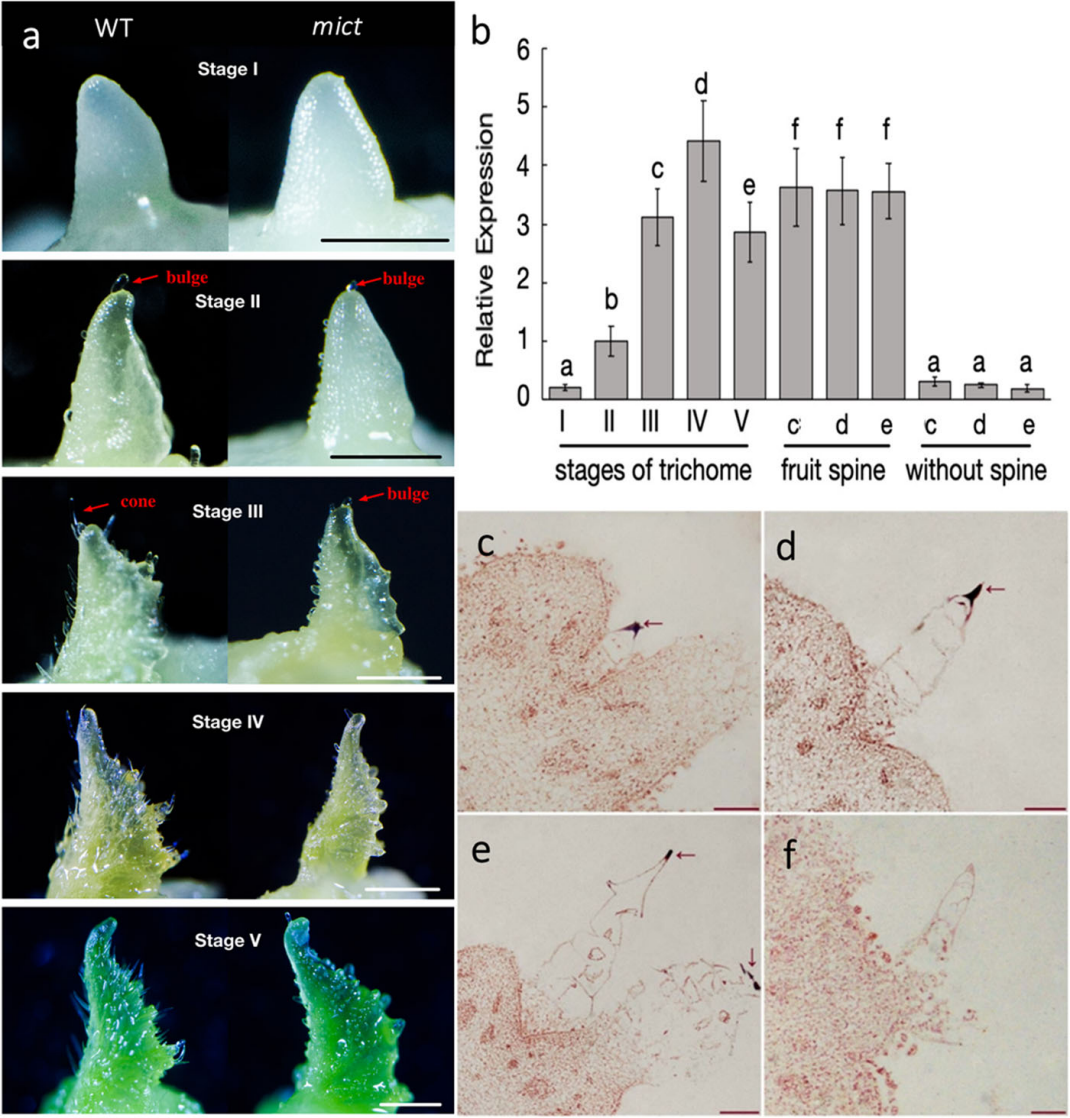
Gene expression analysis of *mict* mutants and WT cucumber plants. **a** Trichome morphological observations in the 5 stages on the first leaf at 24 h, 32 h, 40 h, 48 h, and 56 h after germination. **b** qRT-PCR assay of *Mict* expression in different organs. Panels (**a**–**f**) indicate significant differences by Tukey’s multiple comparisons test with *P* < 0.05 between different samples. (**c**–**f**) RNA in situ hybridization of *Mict* in WT, with (**f**) showing the sense-probe and (**c**–**e**) as the antisense-probe and fruits at 1 cm, 1.5 cm, and 2 cm. qRT-PCR results are shown in (**b**). Scale bars indicate 1 mm in (**a**) and 200 μm in (**c**–**f**)

To understand the in-depth function of *Mict* in the development of trichomes, comparative phenotypic observations of early developmental stages of trichomes were carried out (Fig. 2a).

The most apparent differences in trichomes between the wild-type and *mict* mutant occurred from Stages III to V, where the mutant trichomes maintained a bulge shape without cell division or elongation that occurred in the *wild-type* (Fig. 2a). Therefore, *Mict* regulates trichome development at early stages via its function in cell division or elongation.

We further examined the spatiotemporal expression pattern of *Mict* and revealed that *Mict* mRNA levels were dramatically elevated during the early trichome developmental stages, initiating from Stage I, sharply increasing at Stage II, and peaking at Stage IV while decreasing slightly in Stage V (Fig. 2b). *Mict* was highly expressed in the fruit spine and barely expressed in tissues without the spine (Fig. 2b). Noticeably, in situ hybridization showed that *Mict* transcripts were specifically detected in the first cell of the multicellular trichome (Fig. 2c–f), which indicated that *Mict* is involved in the early stage of trichome development, particularly in initiating the transformation of trichomes from single-cell bulges to cone-shaped multicellular trichomes.

### Loss-of-function of *Mict* strongly affects global changes in genes involved in flavonoid pathways and cuticle metabolism

To reveal the global effects of *Mict* on leaf trichomes and fruit spine development, transcriptome analysis was performed to identify significantly changed metabolic pathways and putative downstream targets of *Mict*. For the RNA-seq of *mict* and wild-type plants, we collected expanded leaves at the four leaves seedling stage and fruits (the blooming female flower’s ovary). FPKM values were used to calculate gene expression levels and differential expression was defined by statistical parameters (*P* < 0.05 and fold change >2 or < −2). A total of 2548 (leaf) and 711 (fruit) genes exhibited differential expression, including 1367 (leaf) and 481 (fruit) upregulated genes and 1181 (leaf) and 230 (fruit) downregulated genes (Table S1).

**Fig. 3:**
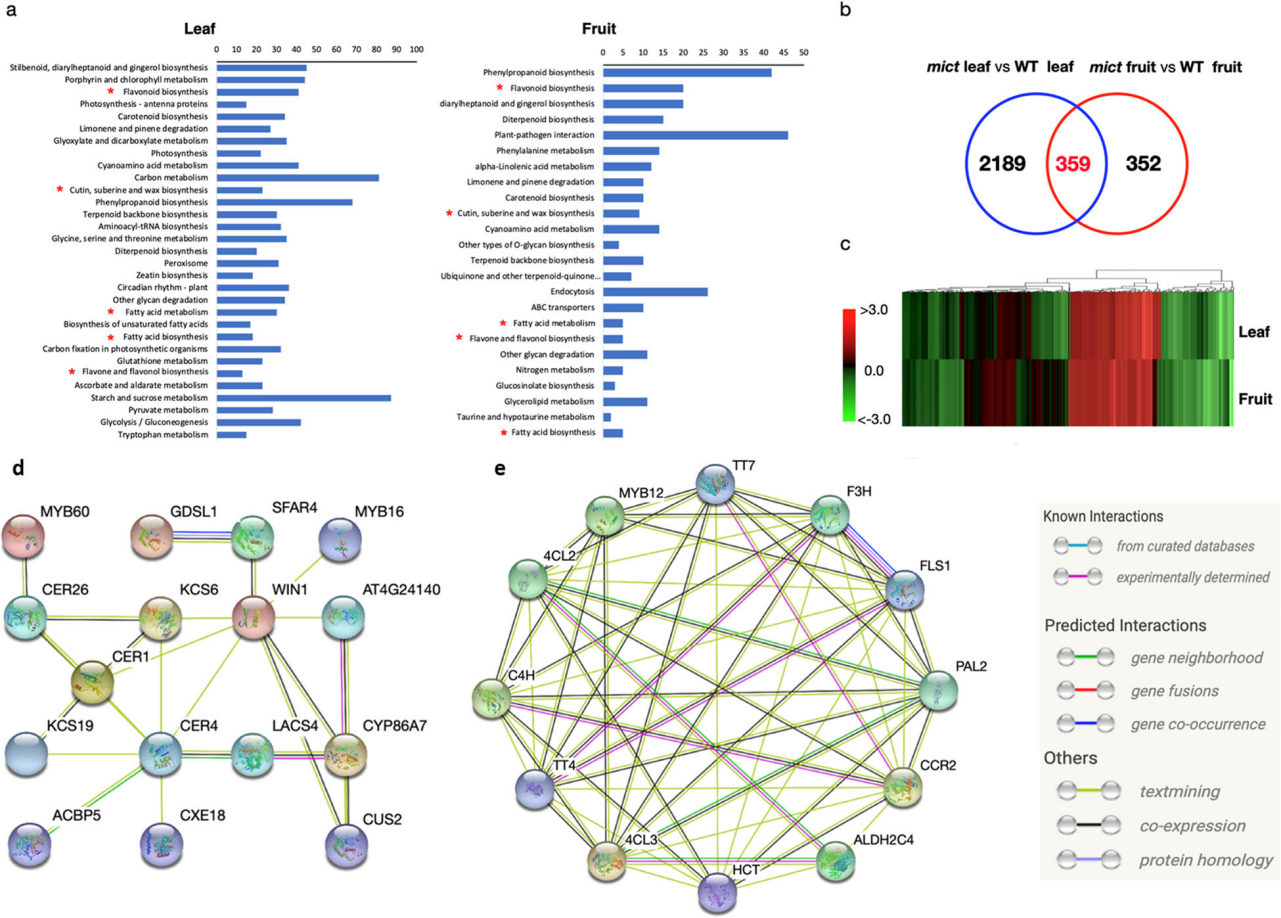
Transcriptome analyses of cucumber leaf and fruit. **a** KEGG pathways that were significantly enriched (Student’s *t*-test *P* < 0.01) in the differentially expressed genes (DEGs) in the leaf or fruit in the *mict* mutant. **b** Venn diagrams of DEGs in the leaves (blue) and fruits (red) in the *mict* mutant. **c** Fold changes of DEGs in the leaf or fruit in the *mict* mutant. A positive number indicates that the gene expression level is upregulated and a negative number indicates that the gene expression level is downregulated. **d** Homologs that were associated with cuticle component biosynthesis. **e** Homologs that were associated with flavonoid biosynthesis. The links between proteins in (**d**) and (**e**) signify the various interaction data supporting the network and are colored by evidence type in the right inset

An analysis of Kyoto Encyclopedia of Genes and Genomes (KEGG) pathways showed that differentially expressed genes (DEGs) were significantly enriched in flavonoids, fatty acids, and cuticle biosynthesis metabolic pathways in both the leaves and fruits (red asterisks in Fig. 3a, and Table S1). Venn analysis identified a common set of 359 DEGs between the leaves and fruits, and heatmap analysis showed that they showed consistent expression patterns between these two tissues (Fig. 3b, c). STRING analysis^18^ was used to predict the functional association networks of the homologs to *Arabidopsis* among these 359 DEGs, which uncovered two arrays of functionally related genes that were closely associated with epicuticular cuticle formation, including waxes, cutin, and flavonoids (Fig. 3d, e). This was in agreement with previous reports of the components of cuticles in plants^8^.

### Compositions of wax and cutin are largely changed in *mict* leaves and fruits

Gas chromatography-mass spectrometry (GC-MS) analysis was carried out to characterize the chemical composition of the leaf and the enzymatically isolated cuticle from *mict* fruit. Overall, compared with wild-type, the abundance of cutin monomer was increased approximately 2-folds in both the *mict* leaf and fruit. The significant increase in the amounts of fatty acids and midchain hydroxylated fatty acids in the leaf, and fatty acids and terminal hydroxylated fatty acids in the fruit mainly contributed to the accumulation of cutin monomers (Tables S2 and S3).

The total waxes in *mict* were reduced to 23.66% (leaf) and 53.33% (fruit) of the values in wild-type. The significant reductions in the amounts of alkanes contributed to reduced wax compounds in the *mict* leaf (Tables S4 and S5). The concentration of fatty acids, alcohols, and alkanes changed little in the *mict* fruit, and the reduction in total wax was mainly caused by the significant reduction in the concentrations of unidentified monomers. The cuticle component differences in the leaf were more significant than in the fruit, which was consistent with the gloss measurements (Fig. 1e). These metabolic analyses clearly indicated that the loss-of-function of *Mict* also affects the leaf and fruit cuticle composition biosynthesis.

### The profiles of flavonoids and lipids are altered in *mict* leaves and fruits

To further reveal the details of the metabolic changes, we performed a comparative metabolomic analysis in leaves and fruits of *mict* and wild-type plants using UHPLC-Q-TOF-MS. A total of 129 and 162 compounds with known structures were identified from the leaves and fruits, respectively. Among those, levels of 71 and 80 compounds were significantly different between the wild-type and *mict* mutant in leaves and fruits (*P* < 0.05), respectively (Table S6).

**Fig. 4:**
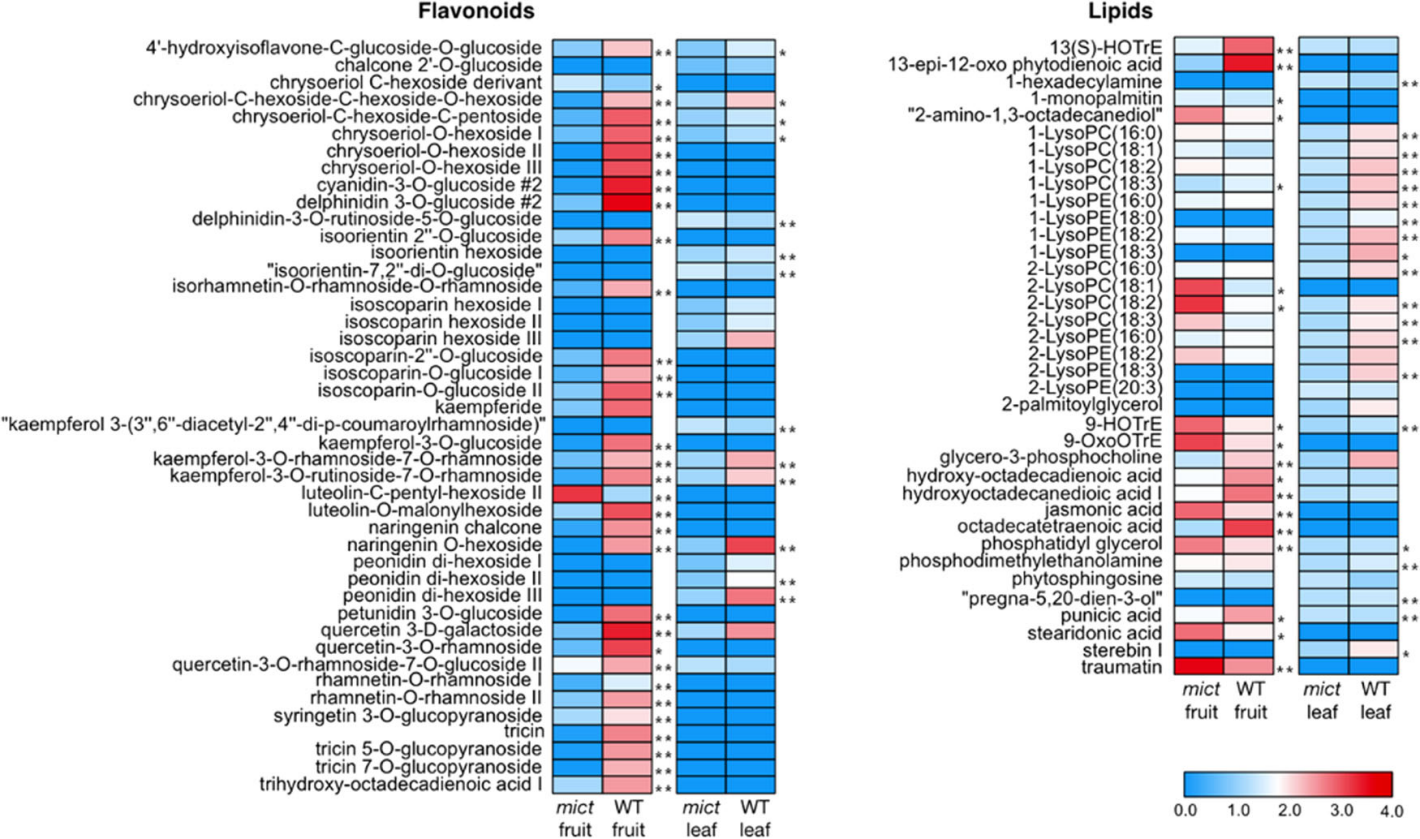
Heatmap of metabolite changes in the fruits and leaves of WT (cucumber line 06-1) and *mict* mutant (06-2). Ratios of fold changes are given by shades of red or blue colors according to the scale bar. Ratios were calculated by taking the logarithm of the mean values from four biological replicates. Student’s *t*-test: ^*^P < 0.05; ^**^P < 0.01

The most notable metabolomic changes were observed in flavonoids and lipids (Fig. 4). In *mict* leaves, 70.37% and 14.81% of detected flavonoids were decreased and increased, respectively. In *mict* fruits, 80.4% and 15.22% of detected flavonoids were decreased and increased, respectively. For lipids, in *mict* leaves, 69.23% and 30.77% of detected lipids were decreased and increased, respectively. In *mict* fruits, 36.54% and 63.46% of detected lipids were decreased and increased, respectively.

This metabolomic results suggest that *Mict* significantly affects lipid and flavonoid metabolic pathways.

### Loss-of-function of *Mict* downregulates the expression of an array of genes involved in flavonoid and cuticle metabolism

**Fig. 5:**
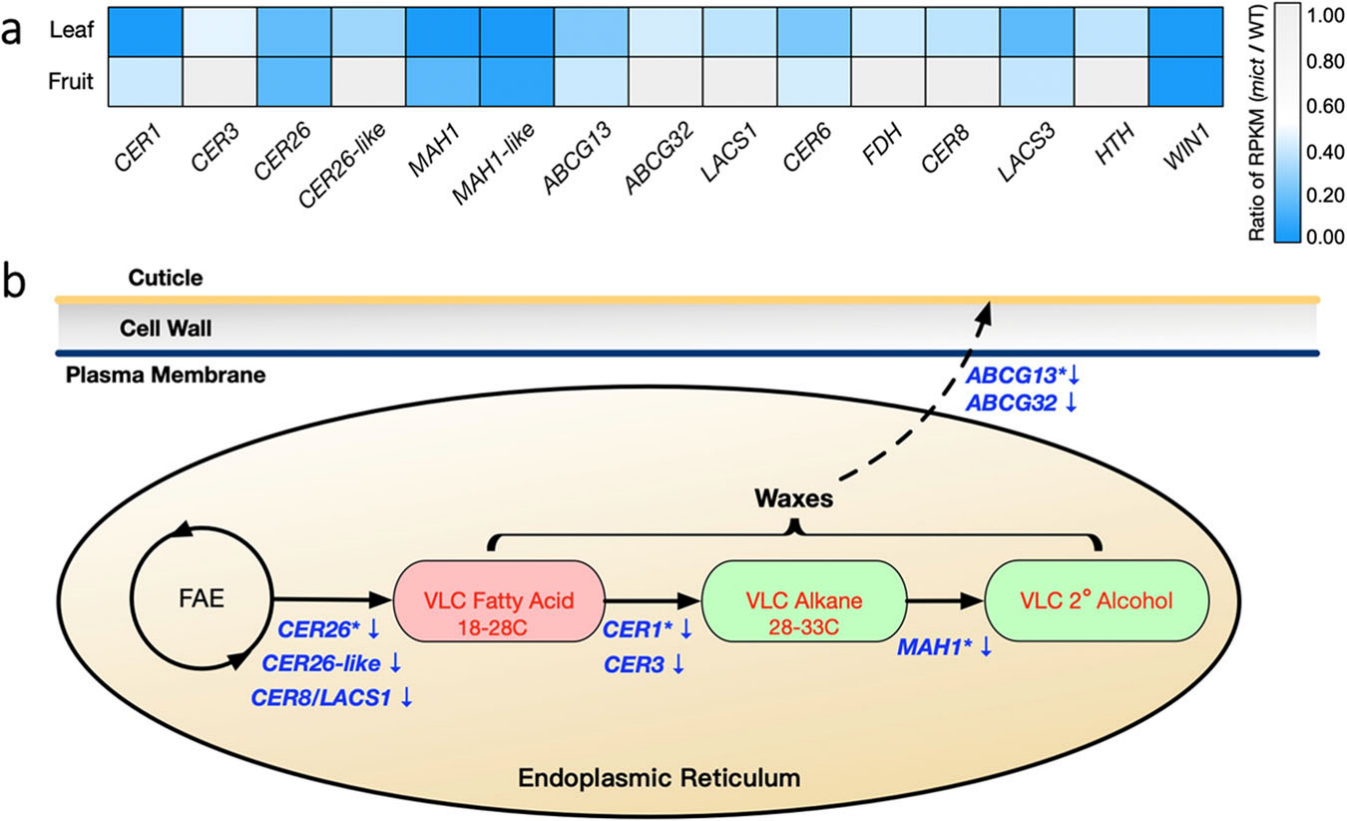
Correlation analysis of the transcriptome and cuticle components. **a** DEGs involved in the cuticle-forming pathways in the leaf and fruit. **b** Simplified diagram of genes involved in cuticle biosynthesis. Genes (blue text) are described in the article. Red text denotes compound classes that are typically observed in wax components

We performed a metabolite-gene correlation analysis to further identify the association of flavonoids and cuticle metabolism pathways with the putative *Mict* target genes. Among the DEGs, 15 putative cucumber orthologues of reported genes involved in cuticle formation in *Arabidopsis* showed significant downregulation in *mict* mutants (Fig. 5a). We further performed a qRT-PCR assay to test the expression levels of these genes in trichomes removed from leaves and fruit peels, which were in agreement with the cuticle composition analysis. We found that although expression of *MAH1-like* and *LACS3* were not significantly changed, the other cuticle-related genes were downregulated in trichomes removed from *mict* leaves (Fig. S1). In fruit peel, except for *CER6* and *HTH*, genes related to the cuticle were also downregulated in *mict*, which was nearly consistent with the transcriptome data (Fig. S1).

**Fig. 6:**
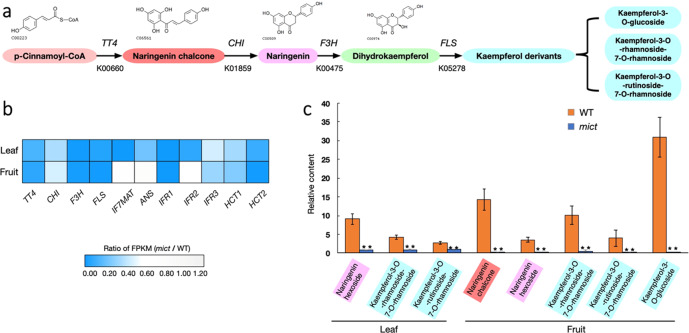
Correlation analysis of transcriptome and flavone compounds. **a** Simplified diagram of part of the flavonoid biosynthetic pathway. TT4, transparent testa 4; CHI, chalcone isomerase; F3H, flavanone 3-hydroxylase; FLS, flavonol synthase. **b** DEGs involved in the flavone biosynthesis pathway in the leaf and fruit. **c** The relative abundance of metabolites related to the pathway in the leaf and fruit. The background color of the compound and its derivatives are consistent with (**a**). Error bars indicate SD (*n* = 3). Student’s *t*-test: ^*^P < 0.05; ^**^P < 0.01

According to the metabolomic and transcriptomic results, flavonoid metabolism was largely hampered in the *mict* mutant, particularly at the step catalyzing p-Cinnamoyl-CoA to kaempferol derivates (Fig. 6a). Moreover, the contents of naringenin chalcone, naringenin, and kaempferol derivates were substantially lower than that of the wild-type (Fig. 6c). Accordingly, the expression of the four key enzymes (i.e., *TT4*, *CHI*, *F3H*, and *FLS1*) that catalyze the above processes was repressed in the *mict* mutant. Other important genes, such as *ANS*, *IFR*, and *HCT*, were also downregulated to varying degrees in the *mict* mutant (Fig. 6b), implying that the flavonoid and flavone biosynthesis pathways are defective if *Mict* is dysfunctional.

### *Mict* positively regulates the expression of *CsTT4*, *CsFLS1*, *CsCER26*, and *CsMYB36* by binding to their promoters

**Fig. 7:**
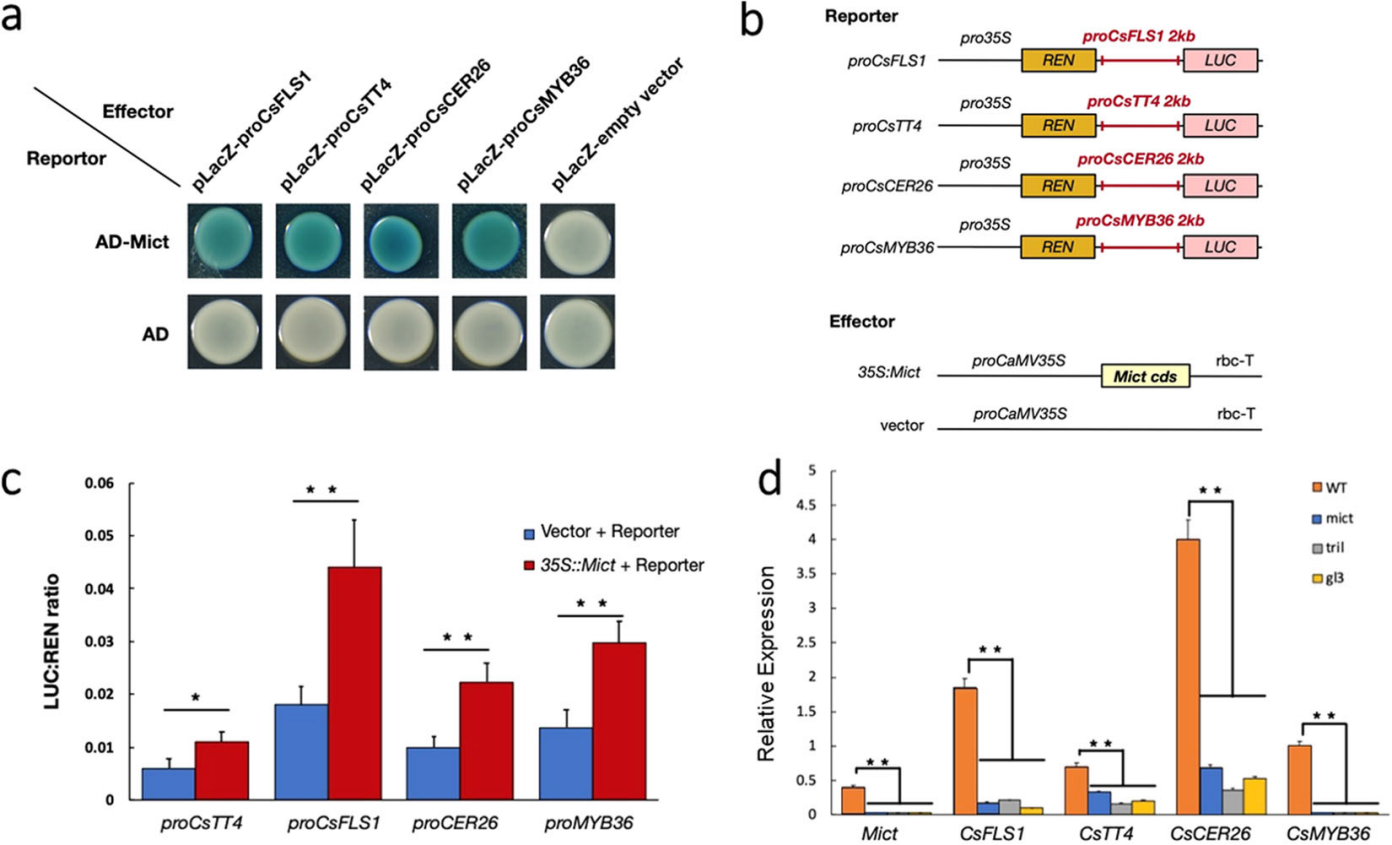
*Mict* activates the expression of *CsTT4*, *CsFLS1*, *CsCER26*, and *CsMYB36*. **a** Yeast one-hybrid assay of protein–DNA interactions. AD, pB42AD. Empty vectors were used as negative controls. Three biological replicates were performed. **b** Structure of the gene constructs being used. **c** Schematic diagram of the reporter and the effector used in the LUC assay showing interactions between *Mict* and the promoters of *CsTT4*, *CsFLS1*, *CsCER26*, and *CsMYB36*. **d** Relative expression of *Mict* and the four candidate genes in the wild-type and *mict*, *tril*, and *gl3* mutants. Error bars indicate SD (*n* = 4). Student’s *t*-test: ^*^P < 0.05; ^**^P < 0.01

Based on the multi-omic results that loss-of-function of *Mict* had a major impact on cuticle component metabolism (Fig. 4) and our finding that the downstream genes may contribute to the abnormal metabolic phenotype (Figs. 5 and 6), we examined whether *Mict* could interact with the promoter of the revealed downstream target genes. We used a yeast one-hybrid assay to test the interaction between *Mict* and the promoters of 47 putative downstream target genes that are associated with the flavonoid and cuticle pathways (out of the 359 DEGs). The results showed that *Mict* could physically interact in vitro with four of these promoters (Fig. 7a), namely, *CsTT4*, *CsFLS1*, *CsCER26* (*Csa2G147920*), and *CsMYB36* (*Csa2G352940*). Meanwhile, co-infiltration of 35S:*Mict* and promoter-LUC constructs resulted in significant transactivation of the promoters of the four tested putative *Mict* targets (Fig. 7b, c). To further explore the *Mict* binding region in the promoters, we segmented the promoters into four 500 bp long fragments (Fig. S3a). The results of yeast one-hybrid assay suggest that the *CsFLS1*, *CsCER26*, and *CsMYB36* binding sites are located in the −2000 to −1500 bp regions of the promoter. For CsTT4, the binding site is located in the −500 to 0 bp region in the promoter (Fig. S3b). In addition, these four genes were also significantly downregulated in the other two trichome mutants (*trichome-less*(*tril*) and *glabrous 3*(*gl3*)), which exhibit recessive epistasis toward *mict* and show a similar phenotype to the *mict* mutant with bright leaves and reduced wax upload (Fig. 7d).

To further confirm the biological functions of *CsTT4*, *CsFLS1*, and *CsCER26*, we created *CsTT4*, *CsFLS1*, and *CsCER26* overexpression *Arabidopsis* transgenic lines. The naringenin chalcone and kaempferol-3-O-glucoside contents were significantly increased in the *oxCsTT4* and *oxCsFLS1* transgenic plants, respectively (Fig. S2a, b), which suggests that *CsTT4* and *CsFLS1* are functionally conserved in the flavonoid biosynthesis pathway. The *CsCER26*-overexpressing plants showed a glossy phenotype compared with the wild‐type plant stem (Fig. S2c), which was consistent with the *AtCER26*-overexpressing *Arabidopsis* plants^13^. Taken together, these results suggest that *Mict* likely affects flavonoid and cuticle metabolism as a direct action on the gene expression of its target genes and is associated with epidermal cell development and patterning.

## Discussion

Based on previous studies, the determination and morphogenesis of the multicellular trichomes of cucumber are regulated by HD-ZIP transcription factors^5,15–17,19,20^, which show differences from unicellular trichomes. The known genes related to the cucumber trichome (spine) development are *tril*, *mict,* and *Tu* (*tuberculate fruit*). *Mict* mediates the morphogenesis of trichomes, and disruption of *Mict* makes most trichomes remain in the single-cell bulge state. In this study, evidence from our multi-omic analysis shows that *Mict* acts as a regulator in the very beginning phase to turn on an array of biological processes related to trichome development, directly or indirectly. Although the functions of *Mict* target genes need to be tested in further research, our results indicate that *Mict* can activate genes involved in flavonoid and cuticular lipid biosynthesis. The main purpose of this study was to identify the connection between transcriptional regulation and metabolic pathways in trichome development via multi-omics analysis and provide evidence to support that *Mict* regulates metabolic pathways (e.g., flavonoids and cuticle) both directly and indirectly.

### *Mict* turns on the regulatory network at the beginning of trichome development

Based on our spatial-temporal expression analysis results, *Mict* is highly expressed at Stage III and Stage IV and is reduced at Stage V, implying that *Mict* functions at a particular stage during trichome development in leaf. Moreover, transcription of *Mict* can hardly be detected in any other organs except fruit spines, which implies that *Mict* acts as a regulator in the very beginning of trichome initiation, modulating a large number of downstream target genes for a transient period. Then, the regulated genes continually promote processes, such as cell division and secondary metabolite biosynthesis, to promote proper trichome development. Other evidence that *Mict* acts as a turn-on switch is that *Mict* is accurately detected in the top cell of the multicellular trichome, inducing successive trichome cells to develop spontaneously after *Mict* activates the regulatory network in the top cell. The expression of transcription factors (e.g., *CsWIN1*, *CsMYB6*, and *CsMYB36*) is absent in *mict* mutants, which implies complex interactions between transcription factors are involved in the transcript regulatory network. As a result, the *mict* mutant shows a defective phenotype in wax biosynthesis. In this study, we narrowed the *Mict* binding sites into a 500 bp region in the promoter of the four downstream genes. Common cis-element analysis of these four promoters suggested that only light response elements were identified in all four promoters (Fig. S3c). Taken together, *Mict* might bind novel elements in the promoters that specifically regulate trichome development, which merits further investigations.

### *Mict* regulates cuticle metabolism in epidermal cells

The plant cuticle is an extracellular lipid structure deposited on the aerial surfaces of organs to protect the plant against environmental stresses^21^. Cuticles are complex mixtures of VLCFAs, with chain lengths of more than 26 carbons. In mutant leaf, the wax composition with more than 26 carbons changed significantly compared to that of WT. However, in mutant fruit, similar significant changes in wax components were lacking. Therefore, the change in VLCFA contents might contribute to observed phenotypes between the two organs. Transcriptomic study revealed significant DEGs involved in fatty acid and cuticle biosynthesis pathways, and led us to examine the cuticle’s wax composition. In combination with metabolomics data, we constructed a schematic diagram to explain wax changes, such as VLCFAs, alkanes, and secondary alcohols, in *mict* mutants (Fig. 5b). Downregulation of *CER26* and *CER26-like* indicated the elongation of 30 carbons or more was blocked in the *mict* mutant^13^. Downregulated *CER8* in the *mict* mutant caused the lacing of alkanes and redundancy of free fatty acids^22^.

Moreover, low expression of *CER1* and *CER3*, which act together to catalyze the formation of alkanes from VLCFA-CoA, also explains the lack of alkanes in the *mict* mutant^23^. *MAH1*, a midchain alkane hydroxylase that catalyzes secondary alcohol formation, was downregulated and related to the absence of 2-C18:OL in the *mict* mutant^24^. In addition, downregulated *ABCG13* and *ABCG32*, functional ABC transporters involved in wax component transport, may also influence the transport of wax from the plasma membrane (PM) to the exterior of the cell^25,26^. In summary, the downregulated expression of the genes mentioned above could partly explain some of the compound changes in cuticle biosynthesis pathways, which means that *Mict* positively regulates the downstream genes directly or indirectly to control these pathways in cucumber leaves and fruits. These results are consistent with the mutation of genes related to trichomes, such as *GL1*, *MYB16, MYB106*, and *CER6*, which can also affect cuticle formation in *Arabidopsis*^4,27–29^.

Interestingly, a recent report found that the specific cuticular wax composition varies between different epidermal cell types, i.e., trichome and pavement cells^30^. However, in this context, it is remarkable that *Mict* has much stronger expression in trichome cells than in other types of cells, leading us to speculate that *Mict* regulates the specific cuticular wax components. Notably, *CER26* has much stronger expression in trichome cells than in other epidermal cells in *Arabidopsis*^13^, making it reasonable that *Mict* positively regulates *CsCER26* to synthesize the trichome-specific cuticular wax composition. It is technologically challenging for us to isolate multicellular trichomes from cucumber leaves, limiting an accurate examination of the trichome-specific cuticular wax composition. In the future, we believe that identification of the trichome-specific cuticular wax composition could reveal the function of *Mict* in cooperative regulation of trichome development and material metabolism.

### *Mict* regulates flavonoid metabolism in trichome development

The trichome is regarded as a bio-factory of secondary metabolites in plants. The advantages of trichome-specific secondary metabolite biosynthesis could be maximizing the protective effects while minimizing the harm of their overaccumulation. Flavonoids are frequently used as pigments and involved in response to the environment and developmental processes^31^. Transcriptional control of the structural genes in the flavonoid biosynthetic pathway has been most extensively studied in plants. In our research, *Mict* was shown to regulate catalytic enzymes, such as *CsTT4* and *CsFLS1*, which participate in flavonoid biosynthetic pathways. The expression of catalytic enzymes usually determines the content of metabolites as they are always located in the downstream regulation area. *FLS* is a key enzyme in flavonoid biosynthesis^32^, and TRANSPARENT TESTA (TT) family members were found to work as transcription factors or enzymes in flavonoid biosynthesis pathways in *Arabidopsis*. Among those, CHALCONE SYNTHASE (CHS), which is encoded by the *TT4* gene, catalyzes the first step of flavonoid biosynthesis for producing naringenin chalcone, a common precursor of various flavonoids^33^. In addition, *IF7MAT* (isoflavone 7-O-glucoside-6”-O-malonyltransferase), *ANS* (anthocyanidin synthase), *HCT* (O-hydroxycinnamoyltransferase), and *IFR* (isoflavone reductase) also play key roles in flavone, flavonol, and flavonoid biosynthesis pathways. The aforementioned genes were greatly downregulated in *mict* mutants, suggesting they may participate in the trichome-specific secondary metabolite biosynthesis pathway via *Mict*-mediated regulation. Accordingly, together with the genes–metabolites association analysis in this study, *Mict* may act upstream in a way that activates regulatory networks in trichome morphogenesis and specific metabolic pathways, which ensures that trichomes develop correctly.

### *Mict* activates *CsMYB36* to form fruit wax-powders

The cucumber fruit wax-powder is a thin layer of white powder that surrounds the fruit, and it is mainly composed of VLCFAs (and its derivatives), such as alkanes, aldehydes, and primary and secondary alcohols^34^. Several cucumber mutants show no wax-powder on the fruits, such as *csmyb36*, *tril/csgl3*, and *mict*. It is clear that *tril* exhibits recessive epistasis with *mict*, as *mict* does to *csmyb36*, which suggests that *CsMYB36* is the main regulator of wax-powder formation. In this study, we found *that Mict* could bind to the *CsMYB36* promoter and stimulate its transcription. This result explains the absence of wax-powder on *mict* and *tril* fruits. Accordingly, the interaction between *Mict* and *CsMYB36* directly bridges trichome development and wax-powder formation, and it provides us with a better understanding of the significance of trichome cell differentiation.

In this study, we identified the metabolic changes related to leaf trichome and fruit spine development in cucumber, and linked flavonoid and cuticle metabolism with transcriptional regulation. However, there are still many metabolites that we could not associate them with corresponding genes due to the limited cucumber molecular pathway databases. Fortunately, in recent years, with the rapid advances in trichome-related gene identification in cucumber, we believe our results will provide important clues to discover biochemical mechanisms in cucumber and reveal the molecular regulatory network involved in trichome development.

## Materials and methods

### Plant materials

The wild-type is cucumber (*Cucumis sativus*) North China type inbred line 06-1. The spontaneous *mict* mutant line 06-2 from 06-1 was grown under standard water, fertilizer management, and pest control with appropriate management in the glasshouse of Shanghai Jiao Tong University in Shanghai. Tobacco (*Nicotiana benthamiana*) and *Arabidopsis* were grown at 22 °C in 24 h continuous light indoors.

### Gloss levels measurement

Fully expanded leaves and mature fruits of cucumber were samples for gloss levels measurement. The equipment we used is a Novo Curve Gloss Meter (RHOPOINT, England). Each measurement was repeated four times.

### Nucleic acid extraction and qRT-PCR

Genomic DNA was extracted from the expanded leaves using the CTAB reagent. Fully expanded leaves and ovaries of female flowers were sampled on the day before flowering for RNA and were frozen in liquid nitrogen immediately and stored at −80°C. The RNA extraction, cDNA reverse transcription, and qRT-PCR assays were performed as reported previously^35^. The gene-specific primers used for qRT-PCR were designed using Primer 3 software. Cucumber Actin3 and *UBIQUITIN* were used as internal controls to normalize and verify the expression data^36,37^. Three biological replicates were used per gene. Each qRT-PCR experiment was performed with three technical replicates. The gene-specific primers are listed in Table S7.

### Materials for RNA-seq

Total RNA was extracted from fully expanded leaves and ovaries of female flowers on the day before flowering. RNA-Seq for comparative transcriptomic analyses of the two phenotypes was performed with three biological replicates. The library was constructed and sequenced using a BGISEQ-500 by the Beijing Genomic Institution (BGI, China). The genomic DNA was removed with two digestions with amplification grade DNase I (Epigenetics, USA). Then, the material was treated as previously reported^36,37^. The clean data have been uploaded to the China National GeneBank (CNGB) (Project ID: CNS0230824, CNS0230826, CNS0230828, CNS0230830, CNS0230832, CNS0230834, CNS0230836, CNS0230838, CNS0230840, CNS0230842, CNS0230844, and CNS0230846).

### In situ hybridization

Young cucumber fruits at different stages of growth (1 cm, 1.5 cm, and 2 cm) were fixed with FAA. In situ sense and antisense probes were amplified with gene-specific primers with SP6 and T7 RNA polymerase-binding sites, respectively. Sample fixation, embedding and pretreatment of sections, hybridization, and immunological detection were performed as described previously^38^. Primers of antisense and sense probes are given in Table S7.

### Bioinformatic analysis

We conducted transcriptome profiling experiments using the DGE approach^39^. Samples were collected from the ovaries of female flowers on the day of flowering and fully expanded leaves from the wild-type and *mict* mutant. Venn tools (Oliveros, 2007–2015) was used for Venn diagram analysis. To identify the homolog in *Arabidopsis*, we used the cucumber protein IDs^40^ to batch query the *Arabidopsis* proteins (TAIR10) using BLASTP on the Cucurbit Genomics Database (http://cucurbitgenomics.org) with an e-value cut-off of 1*e*^−1^. Based on the cucumber homolog in *Arabidopsis*, we predicted the protein interaction network using STRING (https://string-db.org).

### Yeast one-hybrid assay

For the yeast one-hybrid assay, the *Mict* open reading frames (ORFs) were amplified from 06-1 genomic DNA and then cloned into the pB42AD vector (digestion with the EcoRI and XhoI restriction enzymes) using ClonExpress II (Vazyme, Nanjing, China). The 2 kb promoter of CsTT4, CsFLS1, CsCER26, and CsMYB36 from 06-1 were inserted into the vector (digestion with the EcoRI and XhoI restriction enzymes). pB42AD-*Mict* and placZ-CsTT4Pro, placZ-CsFLS1Pro, placZ-CsCER26Pro, or placZ-CsMYB36Pro were co-transformed into the yeast strain EGY48a. The empty vectors were used as negative controls. These transformants were cultivated to confirm whether *Mict* can bind the promoter of the CsTT4, CsFLS1, CsCER26, and CsMYB36^41^. All of the primers used in this study are listed in Table S7.

### Dual-luciferase assay

The assay was carried out as described previously^20^. Briefly, *Mict* ORFs were cloned into pHB driven by the 2*35S promoter to obtain overexpression vectors. Then, the vectors were separately transformed into *Agrobacterium tumefaciens* GV3101 as effectors. The promoters of CsMYB36, CsFLS1, CsTT4, and CsCER26 from 06-1 were cloned into pGREEN 0800 to drive the firefly luciferase reporter gene. The CsMYB36, CsFLS1, CsTT4, and CsCER26 promoter vectors were then co-transformed as described previously^35^. The primers used in this study are listed in Table S7.

### Ectopic expression of *CsTT4*, *CsFLS1*, and *CsCER26* in Arabidopsis

To generate lines overexpressing *CsTT4*, *CsFLS1*, and *CsCER26*, the full-length coding regions of these three genes were amplified using specific primers containing the *BamH*I (5ʹ end) and *Sac*I (3ʹ end) endonuclease sites, and inserted in the reverse orientation into the pHB vector. The recombinant plasmids were transformed into Col (WT) using the floral dip method^42^.

### Metabolite extraction and profiling

The fully expanded leaves and ovaries of female flowers on the flowering day from the wild-type and *mict* mutant were ground into a fine powder. Twenty milligram of fine powder was used for metabolite extraction prior to UHPLC-Q-TOF-MS analysis. As the compound content was calculated by weight, the trichomes and fruit spines were not removed from the samples, which was similar to the transcriptome analysis. The metabolite extraction was analyzed as previously described^43^. All measurements were performed with three replicates.

### Analysis of cuticular wax and cutin chemical analysis

As the compound content is calculated from the surface area, we removed the trichomes and fruit spines from the samples to reduce the area errors caused by the trichomes. For the fruit peels, we collected the peels from fruits at the 10th day after flowering so that the fruits were elongated enough that we could isolate the peel from the trichomes. Cucumber cuticular wax was extracted from fully expanded leaves and the fruit pericarp on the 10th day after flowering. Three independent wild-type (WT) and *mict* mutant lines were analyzed. For cutin analysis, fully expanded leaves and fruits were delipidated in a 10 ml mixture of methanol–chloroform (1:1, v:v) for 2 weeks (solvent changed daily), after which the material was dried, weighed, and used for analysis^44^.

### Statistical analyses

All data are expressed as mean value ± standard deviation (SD) of biological replicates. Statistical significance of the treatment differences was assessed using the Student’s *t*‐test when only two components were compared. In addition, *P* values are from one-way ANOVA (Tukey’s multiple comparisons test, α = 0.05). GraphPad Prism version 8.2.1 for Windows (GraphPad Software, La Jolla, CA, USA) was used for the statistical analyses.

## Supplementary information

Table S7 Primers information used in this study.

Figure S1 qPCR assay of cuticle-related genes.

Figure S2 Analysis of transgenic Arabidopsis.

Figure S3. Promoter analysis of CsCER26, CsFLS1, CsMYB36 and CsTT4.

Table S1 List of genes that are differentially expressed in the wild-type and *mict* mutant.

Tabel S2. Cutin identified in enzymatically isolated cuticles derived from the leaf of *mict* mutant and wild-type cucumber plants.

Table S3. Cuitn identified in enzymatically isolated cuticles derived from the fruit of *mict* mutant and wild-type cucumber plants.

Table S4. Wax identified in enzymatically isolated cuticles derived from the leaf of *mict* mutant and wild-type cucumber plants.

Table S5 Wax identified in enzymatically isolated cuticles derived from the fruit of *mict* mutant and wild-type cucumber plants.

Table S6 Significantly different compounds between the wild-type and *mict* mutant in leaves and fruits.
