# Supplementary material for: Study of micro-trichome (mict) reveals novel connections between transcriptional regulation of multicellular trichome development and specific metabolism in cucumber
Source: Hortic Res. 2021 Feb 1;8:21. doi: 10.1038/s41438-020-00456-0 (PMC7848009; doi:10.1038/s41438-020-00456-0)
Supplement: Supplementary file 2 — Figure S1 qPCR assay of cuticle-related genes. [file 41438_2020_456_MOESM2_ESM.docx]

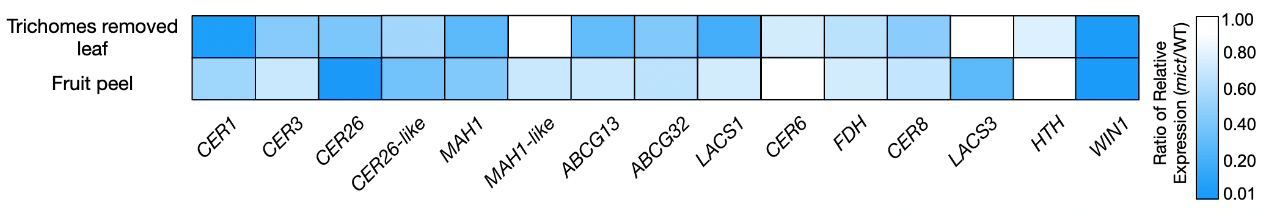
Fig. S1. qPCR assay of cuticle-related genes. Ratios of relative expression are given by shades of white or blue colours according to the scale bar.
