# Supplementary material for: Study of micro-trichome (mict) reveals novel connections between transcriptional regulation of multicellular trichome development and specific metabolism in cucumber
Source: Hortic Res. 2021 Feb 1;8:21. doi: 10.1038/s41438-020-00456-0 (PMC7848009; doi:10.1038/s41438-020-00456-0)
Supplement: Supplementary file 3 — Figure S2 Analysis of transgenic Arabidopsis. [file 41438_2020_456_MOESM3_ESM.docx]

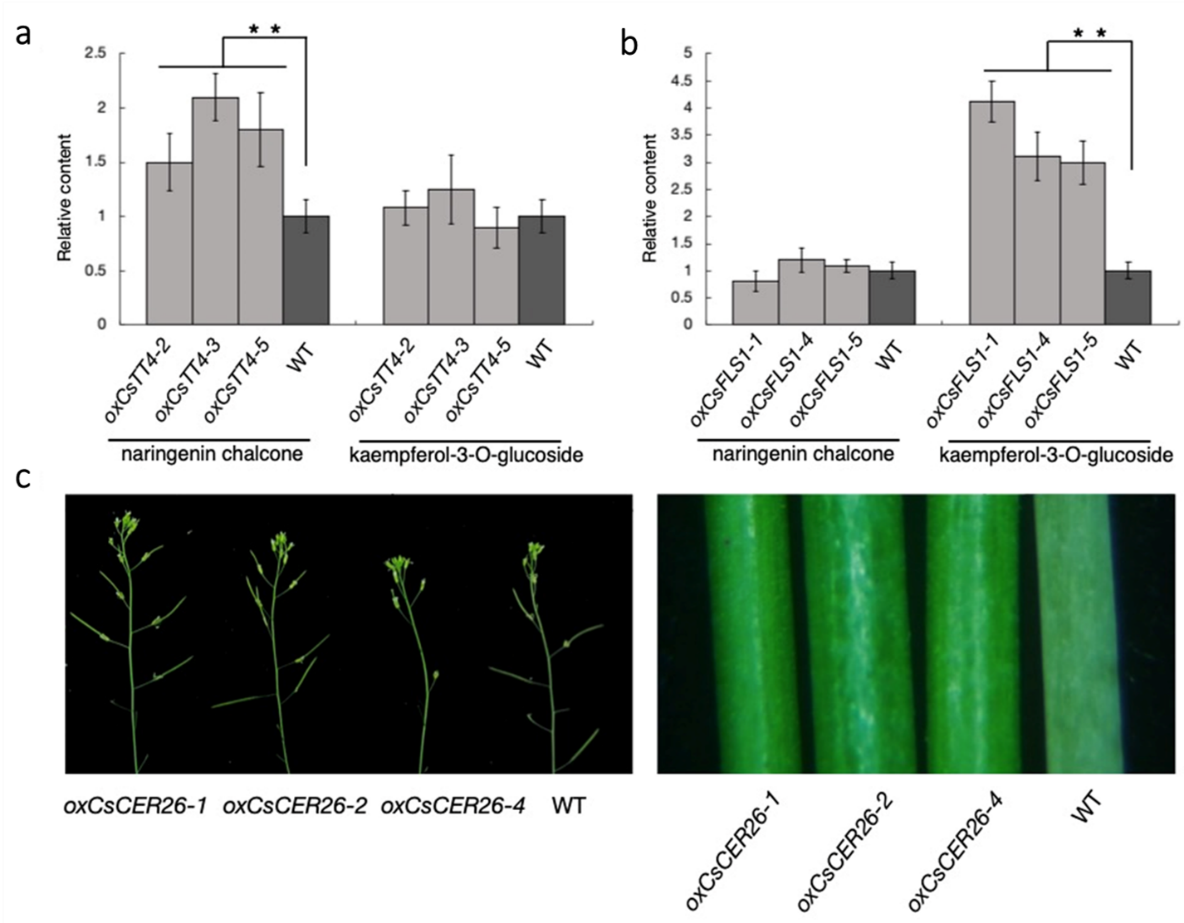


Fig. S2. Analysis of transgenic *Arabidopsis*. The relative content of naringenin chalcone and kaempferol-3-O-glucoside in *oxCsTT4* (a) and *oxCsFLS1* (b) transgenic Arabidopsis seedling plants. Error bars indicate SD (n=3). Student’s t-test: **, P <0.01.(c) Stems from 6–week‐old *CsCER26* overexpressing transgenic Arabidopsis plants (oxCsCER26) (right) and the wild-type (WT) (left).
