# Supplementary material for: Study of micro-trichome (mict) reveals novel connections between transcriptional regulation of multicellular trichome development and specific metabolism in cucumber
Source: Hortic Res. 2021 Feb 1;8:21. doi: 10.1038/s41438-020-00456-0 (PMC7848009; doi:10.1038/s41438-020-00456-0)
Supplement: Supplementary file 4 — Figure S3. Promoter analysis of CsCER26, CsFLS1, CsMYB36 and CsTT4. [file 41438_2020_456_MOESM4_ESM.docx]

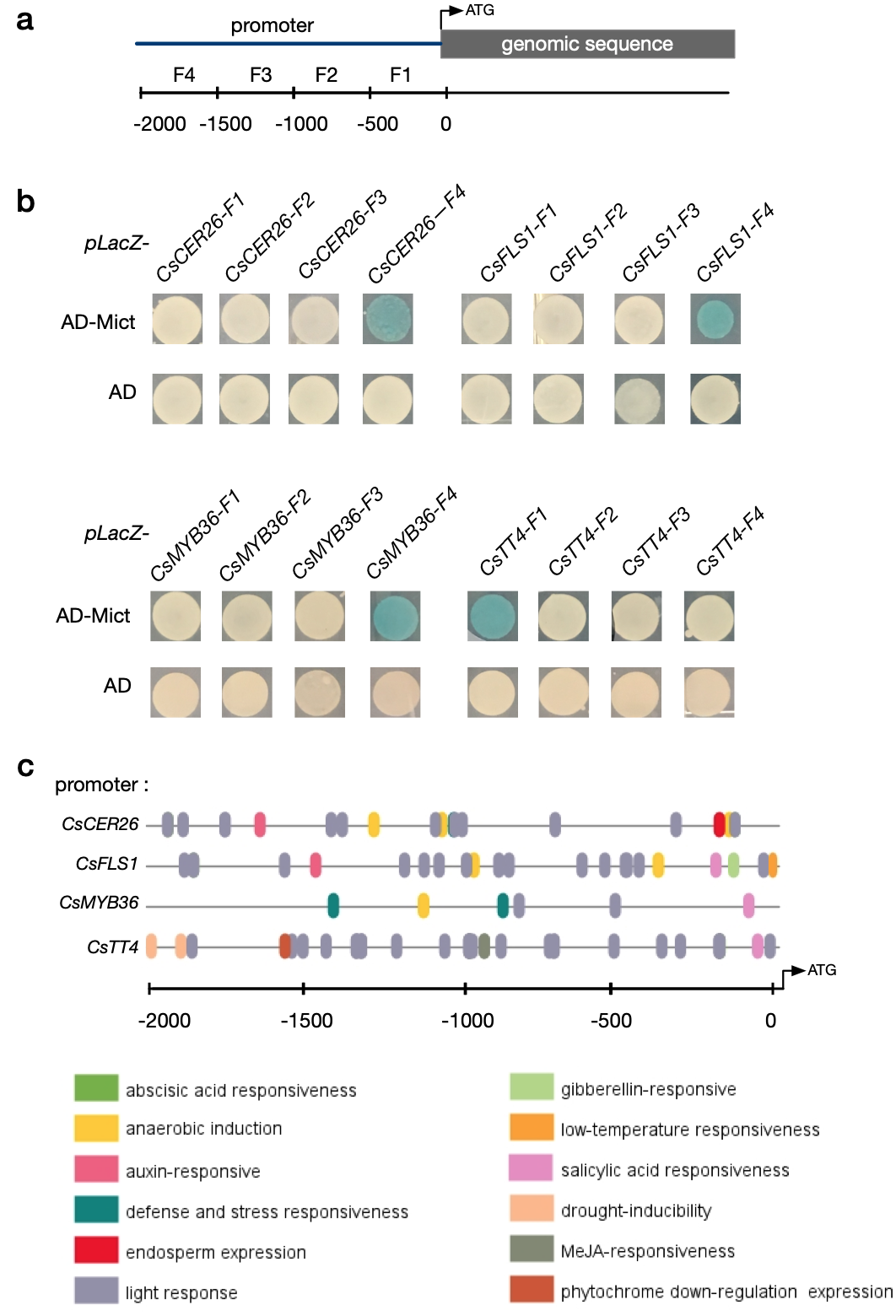


Fig.S3 Promoter analysis of *CsCER26*, *CsFLS1*, *CsMYB36* and *CsTT4*. (a) Schematic diagram of the 500 bp fragments (F1, F2, F3 and F4) in the 2000 bp promoter. (b) Yeast one-hybrid assay showed binding of Mict to F4, F4, F4 and F1 in CsCER26, CsFLS1, CsMYB36 and CsTT4, respectively. (c) Common cis-elements analysis of the promoter of *CsCER26*, *CsFLS1*, *CsMYB36* and *CsTT4.*
